# Fuzzy *C*-Means Clustering Algorithm-Based Magnetic Resonance Imaging Image Segmentation for Analyzing the Effect of Edaravone on the Vascular Endothelial Function in Patients with Acute Cerebral Infarction

**DOI:** 10.1155/2021/4080305

**Published:** 2021-07-14

**Authors:** Jie Yin, Hong Chang, Dongmei Wang, Haifei Li, Aibing Yin

**Affiliations:** Department of Encephalopathy, Qingdao Fifth People's Hospital, Qingdao 266002, China

## Abstract

This paper aimed to discuss the denoising ability of magnetic resonance imaging (MRI) images based on fuzzy C-means clustering (FCM) algorithm and the influence of Butylphthalide combined with Edaravone treatment on nerve function and vascular endothelial function in patients with acute cerebral infarction (ACI). Based on FCM algorithm, Markov Random Field (MRF) model algorithm was introduced to obtain a novel algorithm (NFCM), which was compared with FCM and MRF algorithm in terms of misclassification rate (MCR) and difference of Kappa index (KI). 90 patients with ACI diagnosed in hospital from December 2018 to December 2019 were selected as subjects, who were divided into combined treatment group (conventional treatment + Edaravone + Butylphthalide) and Edaravone group (conventional treatment + Edaravone) randomly, each consisting of 45 cases. The National Institutes of Health Stroke Scale (NIHSS) score and endothelial function index level such as plasma nitric oxide (NO), human endothelin-1 (ET-1), and vascular endothelial cell growth factor (VEGF) were compared before and after treatment between the two groups. The results showed that the MCR of NFCM was evidently inferior to FCM and MRF, and the KI was notably higher relative to the other two algorithms. After treatment, the NIHSS score of the combined treatment group was (9.09 ± 1.86) points and that of Edaravone group was (14.97 ± 3.44) points, with evident difference between the two groups (*P* < 0.05). After treatment, the NO of the combined treatment was (54.63 ± 4.85), and that of Edaravone group was (41.54 ± 5.27), which was considerably different (*P* < 0.01), and the VEGF and ET-1 of combined treatment group were greatly inferior to Edaravone group (*P* < 0.01). It was revealed that the novel algorithm based on FCM can obtain more favorable quality and segmentation accuracy of MRI images. Moreover, Butylphthalide combined with Edaravone treatment can effectively improve nerve function, vascular endothelial function, and short-term prognosis in ACI, which was safe and worthy of clinical adoption.

## 1. Introduction

ACI is a common clinical cerebrovascular disease; together with malignant tumors and heart disease, it is called the “three killers” that endanger human health [1]. According to statistics, in recent years, there are about 2 million new cases of ACI in China each year, and about 1.5 million people die from ACI each year. ACI has the characteristics of rapid onset, high morbidity and disability, and poor prognosis [2]. The tissues of ACI patients undergo ischemia and hypoxia, which in turn causes the body to produce a large number of free radicals. Eventually, the cell membrane is damaged, and in severe cases, the brain tissue will be completely necrotic. ACI can cause different degrees of cognitive dysfunction in patients, such as hemiplegia, aphasia, language dysfunction, hemianopia, and memory loss. Therefore, timely diagnosis and treatment of ACI patients is of great significance. At present, the treatment of ACI patients mainly adopts thrombolytic therapy. However, the actual thrombolysis rate of ACI is very low. The thrombolysis rate in European and American countries is 4.1∼6.3%. The thrombolytic rate in China is only 1.3%. The main reason is that the onset of ACI is urgent, and most patients have missed the best time for thrombolytic therapy when visiting a doctor [3]. There is no effective treatment plan for the treatment of ACI. Edaravone is a commonly used drug in the clinical treatment of ACI patients. Many studies have shown that Edaravone can improve the level of nerve growth factors and reduce inflammatory reactions, which has a certain effect on ACI patients [4]. Butylphthalide promotes the recovery of injured nerves in ACI patients. However, there are few studies on the combination of Butylphthalide and Edaravone in the treatment of ACI.

Medical imaging technology is often used in the diagnosis and treatment of brain diseases. Brain diagnostic imaging technology mainly includes MRI, computed tomography, and ultrasound imaging. Among them, MRI is often used in the diagnosis of brain diseases because of its noninvasiveness, nonionizing radiation, and high resolution. In the process of analyzing cases of brain diseases, doctors need to perform accurate tissue segmentation on brain MRI images to increase the accuracy of disease diagnosis. The original manual segmentation method is time-consuming and inefficient [5]. In recent years, with the development of artificial intelligence and computer-aided technology, a variety of automatic brain MRI image segmentation algorithms have emerged, including threshold method, region growing method, and clustering method. Although the threshold method is simple and fast, it has a poor effect on image segmentation with a lot of noise, uneven gray scale, and low contrast [6]. Although the regional growth method has a wide range of applications, it is very sensitive to the initialization of seed points, and the results are poor in repeatability [7]. The clustering method is used to segment and train MRI images through repeated iterations. The segmentation method of Markov Random Field (MRF) is often adopted in MRI image processing, but its computational complexity is large and parameter estimation is difficult. The fuzzy C-means (FCM) algorithm is a commonly adopted clustering algorithm, and the FCM algorithm is more sensitive to the noise and gray-scale unevenness of MRI images.

In summary, the robustness of the FCM algorithm and the accuracy of brain tissue segmentation needed to be improved. The condition and prognosis of patients with ACI treated with Butylphthalide and Edaravone were unknown, and the mechanism was not clear. FCM algorithm was optimized to improve the accuracy of segmentation of brain tissue by it, which was then applied to the diagnosis of patients with ACI. The patients with ACI diagnosed in hospital from December 2018 to December 2019 were selected as the research objects, to explore the effect of combined treatment of Butylphthalide and Edaravone on neurological function and vascular endothelial function in ACI patients.

## 2. Materials and Methods

### 2.1. Research Subjects and Grouping

A total of 90 patients with ACI who were diagnosed by MRI at XXX Hospital from December 2018 to December 2019 were selected as the research subjects, and the age range was 28–80 years. They were divided into combined treatment group (conventional treatment + Edaravone + Butylphthalide) and Edaravone group (conventional treatment + Edaravone) by random number table, with 45 cases in each group. The inclusion criteria of this study were as follows. I: the first attack, and the onset time was less than 72 h; II: through MRI examination, patients met the diagnostic criteria for cerebrovascular diseases [8]; III: patients older than 18. The exclusion criteria were as follows. I: patients with history of medical and surgical diseases with encephalopathy; II: patients with coma or large-area cerebral infarction; III: patients with dysfunction of the heart, liver, lungs, and kidneys, malignant tumors, and abnormal immune function; IV: patients with drug contraindications; V: patients with cognitive impairment or schizophrenia. The trial process of this study had been approved by the ethics committee of the hospital, and all subjects included had signed the informed consent forms.

### 2.2. MRI Image Segmentation Process

**Figure 1:**
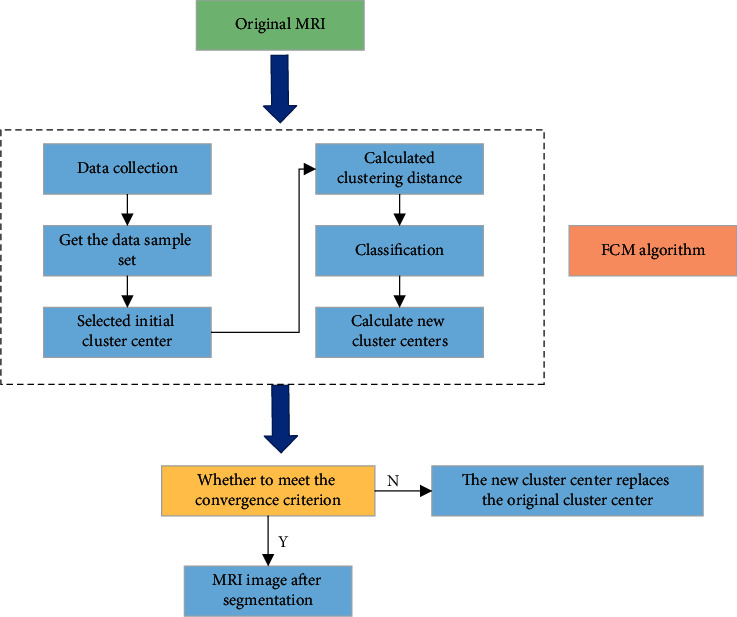
MRI image segmentation process.

For the original MRI image, its subprocess mainly involves the following aspects. First, the initial cluster center of the sample is determined, and the distance between the sample point and the cluster center is calculated. Classification is conducted according to the distance between the sample point and the cluster center. Then, the new cluster center is calculated according to the classification history, and finally whether it meets the cluster convergence criterion is checked, to determine whether the picture is output, as shown in Figure 1.

### 2.3. FCM Algorithm

The FCM algorithm classifies unlabeled data sample points through an objective function based on a certain norm and clustering prototype [9]. It is supposed that there is a given sample set *X*={*x*_1_, *x*_2_,…, *x*_*n*_} ⊂ *A*^*s*^, and *k* samples are randomly selected from this sample set, where *S* refers to dimension of the sample space and *n* represents number of samples. FCM meets the following constraints:

(1) $$\begin{array}{c} \overset{b}{\underset{k=1}{\sum }}\mu_{ki}=1,\\ \overset{n}{\underset{i=1}{\sum }}\mu_{ki}>0,\;1\leq k\leq b,1\leq i\leq n,\\ \mu_{ki}\in \left[0,1\right],\;1\leq k\leq b,1\leq i\leq n, \end{array}$$

and the minimized objective function *G*_FCM_(*U*, *V*)=∑_*k*=1_^*b*^∑_*i*=1_^*n*^*μ*_*ki*_^*m*^*c*_*ki*_^2^ is obtained, where *m* is the fuzzy index, and *m* > 1 in general; *U*={*μ*_*ki*_} is the fuzzy membership matrix of *c* × *n*, and *μ*_*ki*_ is the membership value of the *i*^th^ pixel *x*_*i*_ in the *k*^th^ category; *V*={*v*_1_, *v*_2_,…, *v*_*b*_} represents the matrix *S* × *b*; and *c*_*ki*_=‖*x*_*i*_ − *v*_*k*_‖ represents the distance measure of pixel *x*_*i*_ to the center *v*_*k*_; *b* is the number of clusters that divide *X*. *n* is the number of image pixels.

To minimize the objective function *G*_FCM_, the Lagrange multiplier method is adopted to find the extremum, and a new algorithm is obtained, which is named NFCM. The corresponding *v*_*k*_ algorithm is as follows:

(2) $$\begin{array}{c} v_{k}=\frac{\sum_{i=1}^{n}\mu_{ki}^{m}x_{i}}{\sum_{i=1}^{n}\mu_{ki}^{m}},\;1\leq k\leq b,\\ \mu_{ki}=\frac{1}{\sum_{i=1}^{b}{\left(c_{ki}/c_{i}\right)}^{2/\left(m-1\right)}},\;1\leq k\leq b,1\leq i\leq n, \end{array}$$

and the constraint ∑_*k*=1_^*b*^*μ*_*ki*_=1 is introduced into the objective function of minimization, and the following equation is obtained:

(3) $$\begin{array}{c} L\left(\mu_{ki},v_{k},\alpha \right)=\sum_{i=1}^{n}\left[\sum_{k=1}^{b}\mu_{ki}^{m}{\left\|x_{i}-v_{k}\right\|}^{2}+\alpha \left(1-\sum_{k=1}^{b}\mu_{ki}\right)\right]. \end{array}$$

*α* is the Lagrange multiplier of the constraint condition ∑_*k*=1_^*b*^*μ*_*ki*_=1. To obtain the extreme value of equation (3), it is necessary to obtain the partial derivatives of *L* with respect to the membership degree *μ*_*ki*_ and the clustering center *v*_*k*_, respectively, and set them to zero, that is, (∂*L*/∂*μ*_*ki*_)=0 and (∂*L*/∂*α*)=0. Then, (∂*L*/∂*μ*_*ki*_)=*m*‖*x*_*i*_ − *v*_*k*_‖^2^*μ*_*ki*_^*m*−1^ − *α*=0 and 1 − ∑_*k*=1_^*b*^*μ*_*ki*_=0, and the further available calculation method of *μ*_*ki*_ is as follows:

(4) $$\begin{array}{c} \mu_{ki}={\left(\frac{\alpha }{\left(m{\left\|x_{i}-v_{k}\right\|}^{2}\right)}\right)}^{1/\left(m-1\right)}. \end{array}$$

Constraints are introduced into the calculation method of *μ*_*ki*_, and then *μ*_*ki*_=∑_*i*=1_^*b*^((‖*x*_*i*_ − *v*_*k*_‖^2^)/(‖*x*_*i*_ − *v*_*i*_‖^2^))^1/(*m* − 1)^.

MCR [10] and objective evaluation index KI [11] are adopted to analyze the accuracy of algorithm segmentation. Among them, the calculation method of MCR is MCR=(*M*/*T*), where *M* represents the number of misclassified pixels, and *N* represents the total number of pixels in the image. The calculation equation of KI is KI=(2TP)/(2TP+FP+FN), where TP (true positive) indicates number of samples taken as positive by the model; FN (false negative) represents number of negative samples taken as positive; and FP (false positive) represents number of positive samples taken as negative.

### 2.4. Diagnosis and Treatment of Patients with ACI

All the study subjects were diagnosed by MRI, and the MRI examination was performed by Japan's Toshiba dual gradient scanner (vantage elan MRT-2020, Z1A17Z2063). MRI contrast-enhanced magnetic resonance angiography was performed after routine examination of all patients, and the images were analyzed using GE Advantage Windows workstation.

Routine treatment of cerebral infarction includes symptomatic treatment of blood sugar, blood lipid, and blood pressure. The Edaravone group was treated with Edaravone injection based on conventional treatment. 30 mg of Edaravone was mixed with 100 mL of 0.9% sodium chloride solution and injected twice a day intravenously. The combined treatment group was given Edaravone combined with Butylphthalide treatment based on conventional treatment. 30 mg of Edaravone was mixed with 100 mL of 0.9% sodium chloride solution and injected intravenously. Meanwhile, Butylphthalide soft capsule was taken before meals, 200 mg/time, 3 times/day, every 10d as a course of treatment.

### 2.5. Observation Index and Evaluation Methods

NIHSS mainly included 15 items, and 7 points is taken as the critical value of neural function judgment [12]. The scores of the NIHSS of the two groups of patients before and after treatment were counted. The higher the NIHSS score, the more severe the neurological impairment. The plasma ET-1 level was detected by ELISA. The level of NO in plasma was determined by Griess assay. The level of VEGF in plasma was detected by ELISA.

### 2.6. Statistical Methods

The test data was processed using SPSS19.0 statistical software. The basic data of the patients, age, weight, height, BMI, time of onset, NIHSS score, ET-1, NO, and VEGF levels were expressed as mean ± standard deviation ($\bar{x}$ ± *s*). The *t*-test was adopted to analyze the differences between the groups. The count data of proportions of different genders were expressed as percentages (%), and the *χ*^2^ test was adopted for analysis. *P* < 0.05 was taken as statistically significant difference.

## 3. Results

### 3.1. Analysis of MRI Image Segmentation Effect Based on FCM Algorithm

**Figure 2:**
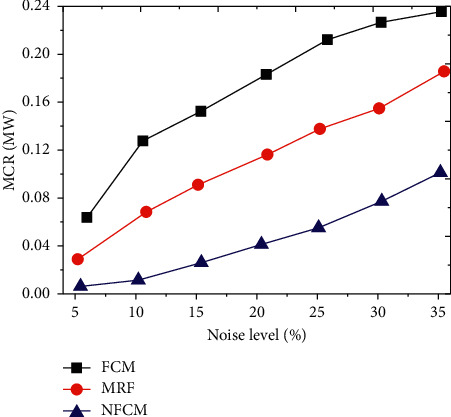
Comparison of ultrasound imaging resolution of different algorithms.

The MCR value of the new algorithm NFCM, FCM algorithm, and MRF algorithm was compared and analyzed under different noise levels. The results are shown in Figure 2. Under different noise levels, as the noise level continued to increase, the MCR values of all algorithms showed an upward trend. The MCR value of NFCM algorithm based on FCM was absolutely inferior to that of FCM and MRF algorithm.

**Figure 3:**
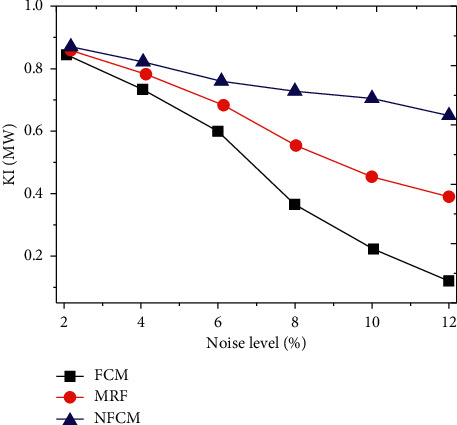
Comparison of ultrasound imaging contrast of different algorithms.

The KI changes of the new algorithm NFCM, FCM algorithm, and MRF algorithm under different noise levels were further analyzed, and the results are shown in Figure 3. Under different noise levels, as the noise level continued to increase, the KI values of all algorithms showed a downward trend. The KI value of the new algorithm based on FCM (NFCM) was evidently higher relative to that of FCM and MRF algorithms.

### 3.2. MRI Imaging of Patients with ACI

**Figure 4:**
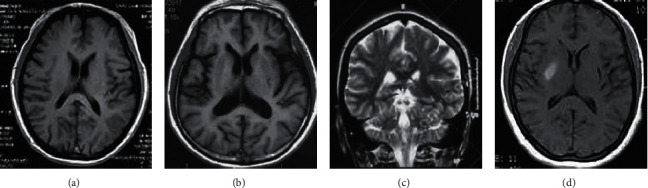
MRI image of patients with ACI. (a) MRI image of normal human brain; (b) MRI image of posterior cortical cerebral infarction; (c) MRI image of cerebellar watershed infarction; (d) MRI image of intracerebral cerebral infarction.

MRI was adopted to diagnose patients with ACI; the results are shown in Figure 4. In contrast to normal human brain MRI images, posterior infarction type cerebral infarction appeared as a bead-like or fused strip-like high signal area. MRI manifestations of posterior internal cerebral infarction were bead-like or fused into a strip of high signal area. MRI manifestations of postcortical cerebral infarction were brief and lateral to the base of the brain toward the pial surface.

### 3.3. Contrast of Basic Data of Two Groups of Patients

The basic data of patients were compared, and the results are shown in Table 1. There is no considerable difference between patients' gender, age, and time of onset, without any notable difference (*P* > 0.05).

### 3.4. Comparison of NIHSS Scores between Groups

**Figure 5:**
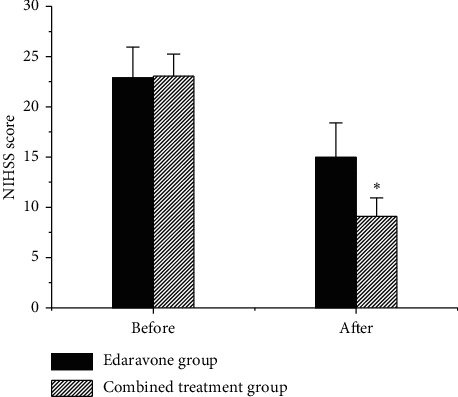
Contrast of NIHSS scores between the two groups (^*∗*^a very substantial difference relative to Edaravone group, *P* < 0.05).

The NIHSS scores of patients were compared, as shown in Figure 5. No substantial difference was shown in the NIHSS scores of patients before treatment (*P* > 0.05). After treatment, the NIHSS scores of the two groups of patients showed a downward trend. The NIHSS score of the combined treatment group was (9.09 ± 1.86) points and that of the Edaravone group was (14.97 ± 3.44) points, which was substantially higher in contrast to the combined treatment group, with notable difference (*P* < 0.05).

### 3.5. Contrast of Vascular Endothelial Function Indexes between the Two Groups

**Figure 6:**
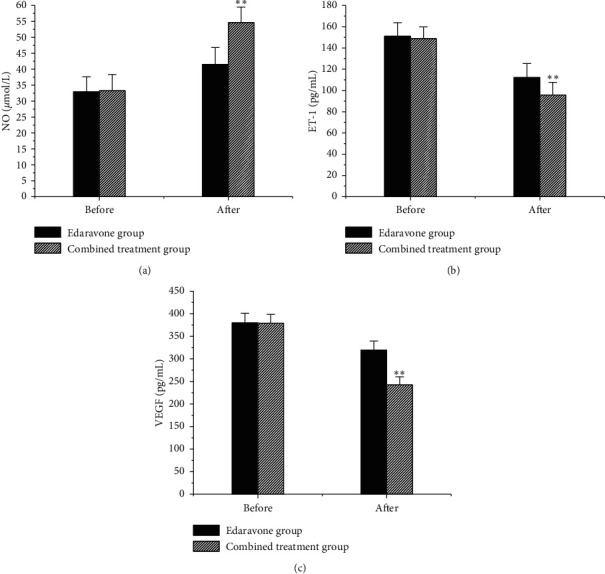
Contrast of vascular endothelial function indexes between groups before and after treatment. (a) Comparison of the blood NO values; (b) comparison of the blood ET-1 values; (c) comparison of the blood VEGF values (^*∗∗*^a very considerable difference relative to the Edaravone group, *P* < 0.01).

The vascular endothelial function indexes (NO, ET-1, and VEGF) of patients were compared, as shown in Figure 6. There was no notable difference in vascular endothelial function indexes NO, ET-1, and VEGF between groups before treatment (*P* > 0.05). After treatment, the NO of the two groups of patients increased. The NO of the combined treatment group was (54.63 ± 4.85) and the NO of the Edaravone group was (41.54 ± 5.27). The NO of the combined treatment group was obviously higher compared to Edaravone group (*P* < 0.01). After treatments, the ET-1 of patients decreased, and the ET-1 of the combined treatment group was obviously inferior to Edaravone group, and the difference was notable (*P* < 0.01). After treatments, the VEGF of patients decreased, and the VEGF of the combined treatment group decreased more than the Edaravone group, and there was a very notable difference between the two (*P* < 0.01).

## 4. Discussion

According to the characteristics of the brain MRI image, the FCM algorithm was used to segment the brain. Based on the FCM algorithm, MRF model algorithm was introduced to increase the accuracy of MRI image segmentation. The research results found that the MCR value of the new algorithm (NFCM) under different noise levels was greater in contrast to the FCM and MRF algorithms, and its KI value was also greater than FCM and MRF algorithm. The research results of Liu et al. [13] showed that when the MRI segmentation result was completely consistent with the ground truth image, the KI value was 1. Moreover, the closer the MRI segmentation result to the ground truth image, the greater the KI value [14]. Therefore, the new algorithm based on the FCM algorithm had a higher accuracy of brain segmentation and a better segmentation effect.

The higher the NIHSS score, the more severe the neurological impairment of the subject [15]. The results of the study showed that there was no evident difference in NIHSS scores between groups before treatment (*P* > 0.05). After treatment, the NIHSS score of the combined treatment group was considerably inferior to Edaravone group (*P* < 0.05), indicating that the combined adoption of Edaravone and Butylphthalide can improve the nerve damage in patients with ACI greatly. Edaravone is currently a commonly used ACI treatment drug in clinic. It has an antioxidant effect [16] and can improve the neurological function of ACI patients [17]. Butylphthalide can reduce brain tissue damage due to its effect on oxidative stress [18], so it is also used in the treatment of ACI patients. It was found that no obvious difference was shown between the vascular endothelial function indexes NO, ET-1, and VEGF before treatment (*P* > 0.05). Moreover, ACI patients had higher levels of ET-1 and VEGF and lower levels of NO before treatment. These results indicated that the vascular endothelial function of ACI patients was severely damaged before treatment. After treatments, the NO of patients increased, and the NO of the combined treatment group was greatly higher relative to Edaravone group (*P* < 0.01). Wang et al. [19] found that Butylphthalide can reduce the content of arachidonic acid, and it can increase the level of NO in patients with ACI, which had the effect of enhancing the activity of antioxidant enzymes, thereby achieving the purpose of anti-brain tissue ischemia. It was also consistent with the results of this work. The results revealed that the ET-1 and VEGF of patients decreased after treatment and those of the combined treatment group were greatly inferior to Edaravone group (*P* < 0.01). Tang et al. [20] found that the adoption of Butylphthalide in patients with ACI can substantially reduce the level of VEGF in patients, which was similar to the results of this work. The above research results showed that when Butylphthalide combined with Edaravone treated ACI, not only can the degree of nerve damage in patients be notably improved, but also the impaired vascular endothelial function can be improved. The reason was further analyzed; adding Butylphthalide based on Edaravone antioxidant may further enhance its antioxidant function. Moreover, Butylphthalide has the function of protecting mitochondria, which apparently improved hemodynamics and reduced the degree of brain tissue damage under hypoxia [21]. In summary, the treatment of ACI with Butylphthalide combined with Edaravone can evidently improve the patient's nerve damage and vascular endothelial function.

## 5. Conclusion

Based on the FCM algorithm, the MRF model algorithm was introduced to increase the accuracy of MRI image segmentation, which was applied to the diagnosis of patients with ACI. The effects of Butylphthalide combined with Edaravone treatment on nerve function and vascular endothelial function in patients with ACI were further analyzed. It was found that the new algorithm based on FCM algorithm had better segmentation effect. When Butylphthalide combined with Edaravone was adopted in the treatment of patients with cerebral infarction, it can greatly improve the patient's neurological impairment and vascular endothelial function. However, there are still some shortcomings in this article. The patients collected in this study are all in the same hospital and the number of cases is small, which has certain limitations. Moreover, in the analysis process, the vascular endothelial function indexes at different time periods after treatment are not tested. In the later work, while increasing the amount of sample collection, the vascular endothelial function indexes at different time periods after treatment will be tested, to further confirm the clinical value of Butylphthalide combined with Edaravone in the treatment of cerebral infarction. In summary, the new FCM-based MRI image quality and segmentation accuracy are higher. Moreover, Butylphthalide combined with Edaravone treatment can effectively improve neurological function, vascular endothelial function, and short-term prognosis in ACI, and it is safe and worthy of clinical adoption.

## Figures and Tables

**Table 1:** Contrast of basic data of patients.

Group	Edaravone group	Combined treatment group	*P*
Male (case (%))	27 (60.00)	29 (64.44)	0.451
Age (year)	60.25 ± 4.82	59.78 ± 8.82	0.273
Height (cm)	167.42 ± 6.93	165.26 ± 9.54	0.366
Weight (kg)	60.47 ± 4.82	59.86 ± 5.39	0.158
BMI (kg/cm^2^)	21.72 ± 0.94	22.31 ± 0.96	0.254
Onset time (h)	5.89 ± 1.26	6.22 ± 1.78	0.151

## Data Availability

No data were used to support this study.
